# Examining implicit metacognition in 3.5-year-old children: an eye-tracking and pupillometric study

**DOI:** 10.3389/fpsyg.2013.00145

**Published:** 2013-03-22

**Authors:** Markus Paulus, Joelle Proust, Beate Sodian

**Affiliations:** ^1^Department of Psychology, Ludwig Maximilian UniversityMunich, Germany; ^2^Ecole Normale Supérieure, Institut Jean-NicodParis, France

**Keywords:** memory, metacognition, implicit, preschool age, eye-tracking

## Abstract

The current study examined early signs of implicit metacognitive monitoring in 3.5-year-old children. During a learning phase children had to learn paired associates. In the test phase, children performed a recognition task and choose the correct associate for a given target among four possible answers. Subsequently, children's explicit confidence judgments (CJs) and their fixation time allocation at the confidence scale were assessed. Analyses showed that explicit CJs did not differ for remembered compared to non-remembered items. In contrast, children's fixation patterns on the confidence scale were affected by the correctness of their memory, as children looked longer to high confidence ratings when they correctly remembered the associated item. Moreover, analyses of pupil size revealed pupil dilations for correctly remembered, but not incorrectly remembered items. The results converge with recent behavioral findings that reported evidence for implicit metacognitive memory monitoring processes in 3.5-year-old children. The study suggests that implicit metacognitive abilities might precede the development of explicit metacognitive knowledge.

## Examining implicit metacognition in 3.5-year-old children: an eye-tracking and pupillometric study

Metacognitive knowledge and abilities, that is, cognition about one's own cognitive activities, play an important role in human cognitive performances (e.g., Hofer and Pintrich, 1997). According to classical models, metacognition consists of a monitoring component, which supervises and evaluates cognitive performances, and a control component, which regulates cognitive activities (e.g., Nelson and Narens, 1990; Kuhn, 2000). Monitoring one's own cognitive activities and using this knowledge to guide future activities play an important role in memory and learning. Several studies have reported positive relations between metacognitive competencies and knowledge, and performances in memory tasks (e.g., Schneider and Pressley, 1997; Renner and Renner, 2001; Dunlosky et al., 2005).

Despite the relevance of metacognition for human performance, little is known about the early development of metacognitive abilities (cf. Sodian et al., 2012). In fact, most developmental studies investigating metacognitive competencies have relied on assessments of explicit metacognitive knowledge and evaluations in school-aged children (e.g., Hofer and Pintrich, 1997; Veenman et al., 2006). These studies suggested that metacognitive (monitoring) abilities do not develop before the school age and undergo a slow developmental progression from early school age to adolescence (for a review see Schneider and Pressley, 1997). Yet, a few studies investigating children's employment of memory strategies to aid memory performance, an indirect measure of metacognitive control, have suggested that some metacognitive abilities might already develop between 4 and 6 years of age (e.g., Sodian et al., 1986; Schneider and Sodian, 1988).

Motivated by recent findings in the animal literature (e.g., Hampton, 2001; Smith et al., 2003; Kornell et al., 2007; Call, 2010), developmental researchers have started to examine the implicit signs/precursors of metacognitive abilities in younger children (e.g., Call and Carpenter, 2001; Balcomb and Gerken, 2008; Gerken et al., 2011). Balcomb and Gerken (2008), for example, relied on an opt-out paradigm to examine early signs of implicit metamemory in preschool children. They presented 3.5-year-old children with a memory task in which they had to learn paired associates. In the test phase, children were first presented with the first of the two items and had the possibility to skip answering this item. The authors reported that the children more often declined items for which they displayed worse memory performance in a subsequent test. The results indicated that the children were able to evaluate their own memory. This study provided first evidence for implicit metacognitive performance in a memory task in children younger than 4 years of age.

Moreover, recently Lyons and Ghetti (2013) presented 3–5-year-old children with a perceptual discrimination task. They showed that the children selectively withhold responses for trials in which they reported uncertainty. This suggests that children are aware of their own knowledge and rely on this evaluation to strategically volunteer or withhold responses. Taken together, these behavioral studies indicate an early developing appreciation of one's own knowledge state in the preschool period.

The present study aimed at further examining implicit metacognitive abilities in 3.5-year-old children, employing an eye-tracking paradigm. Eye-tracking has become a frequently used, nonverbal measure of young children's performances (e.g., Paulus et al., 2011; De Bordes et al., 2012; Elsner et al., 2012a,b; Fawcett and Liszkowski, 2012). In particular, it has been shown to be a suitable method to investigate young children's learning performances (e.g., Johnson et al., 2003; McMurray and Aslin, 2004; Roebers et al., 2010; Paulus and Fikkert, 2012) and, in school-aged children, also metamemory skills (Roderer and Roebers, 2010). Roderer and Roebers (2010) examined 7–9-year-old children's explicit confidence judgments (CJs) in a memory task. Additionally, they recorded participants' fixation time allocation on the CJ scale. The results showed that older children's implicit CJ as measured by their visual fixation differentiated between easy and difficult items. They allocated more looking time to the parts of the CJ scale expressing high confidence for easy compared to difficult or unanswerable items. Moreover, implicit and explicit CJ were strongly related to each other as indicated by correlations between implicit and explicit judgments. This study demonstrates that the analysis of participants' fixation pattern during their CJs in a memory task allowed for an assessment of their memory monitoring. It also shows that in school-aged children implicit and explicit metacognitive processes were related to each other. Thus, the study provides evidence that eye-tracking of looking patterns is sensitive to fast and implicit processes (for reviews see Karatekin, 2007; Gredebäck et al., 2010), and is therefore suitable to study metacognitive processes in young children.

Moreover, recent research has shown that pupil dilations open another window on early cognitive processes already in preschool children (e.g., Gredebäck and Melinder, 2011; Gredebäck et al., 2012). In particular, it has been argued that pupil dilations reflect the intensity of mental processes. Importantly, the subject does not need to be aware of these processes as pupil dilations have been shown to be sensitive to implicit or preconscious processing (Bijleveld et al., 2009; for a recent review see Laeng et al., 2012). A number of studies demonstrated that pupil dilations are a useful measure of memory processes (for a review see Goldinger and Papesh, 2012).

Papesh and colleagues (Papesh et al., 2011), for example, presented adult participants in a study phase with 80 items (40 words, 40 non-words). In a subsequent test phase, participants listened to 160 items and were asked to make old/new judgments by indicating their confidence on a 6-point scale (1: very sure new; 6: very sure old). The authors assessed participants' pupil size during the study and the test phase. The analyses revealed that trials, leading to high confidence ratings, yielded larger pupil size in the study phase than trials, leading to lower confidence ratings. Furthermore, the analyses of the test trials showed that pupil size were larger during correct than incorrect trials, and that larger pupil size were related to high-confidence choices. This shows that besides fixation patterns, changes in pupil size are a sensitive measure to investigate cognitive and, in particular, memory processes. In particular, it provides evidence that pupil dilations are related to memory strength and CJs.

To assess memory monitoring in preschool children we presented children with a paired associate learning task following the design of Balcomb and Gerken (2008). In a learning phase children had to learn animal-object pairs. In a test phase, we presented children with the same animals and assessed their memory of the associated object. More concretely speaking, they were asked to choose the correct item amongst four possible alternatives. Subsequently, they were asked to give an explicit CJ on a 5-point scale. At the same time, we measured their looking pattern to the judgment scale as a measure of implicit memory monitoring. By comparing their explicit answers for correctly and incorrectly remembered items with their (implicit) looking patterns at the scale, we aimed at examining whether or not the 3.5-year-old children would differentiate between correctly and incorrectly remembered items.

Given previous results that pointed to limitations of explicit metacognitive skills in young children (cf. Schneider and Pressley, 1997) we expected no differentiation for correct and incorrect items in the explicit CJ. However, given the evidence on implicit metacognitive abilities in behavioral tasks (e.g., Balcomb and Gerken, 2008) we hypothesized differences in CJs between the items in the implicit measure, that is, children's fixation patterns. More concretely speaking, following the findings of Roderer and Roebers (2010), we expected participants to allocate more looking time to the parts of the CJ scale expressing high confidence for correctly compared to incorrectly answered items. Finally, we analyzed changes in pupil size by comparing children's pupil size for the animal in the learning trials and the presentation of the same animal in the test trials. Given the claims that pupil dilations reflect cognitive processes already in young children (e.g., Gredebäck and Melinder, 2011; Laeng et al., 2012) and given findings of pupil dilations for well-remembered items in adults (e.g., Papesh et al., 2011), we expected to find pupil dilation for items that children were able to answer correctly, but not for incorrectly answered items.

## Methods

### Participants

The final sample included 12 3.5-year-old children (*range:* 42–49 months; 7 boys). 5 additional participants were tested but not included in the final analysis due to refusal to continue the study (*n* = 2) or due to procedural and experimental errors (*n* = 1). Two further subjects needed to be excluded due to answering all items correctly. As we compared answers for correctly vs. incorrectly remembered items within subjects, the data of these two subjects did not allow for such a comparison. The participants were recruited from birth records of the local district administration authority. Children were native German speakers from heterogeneous socioeconomic backgrounds. Informed consent for participation was given by the children's caregivers. Parents received a monetary compensation for travel expenses and the children a gift for their participation.

### Stimuli

The stimulus material followed the design of Balcomb and Gerken (2008) and consisted of 18 item pairs. The material for every pair consisted of line drawings of an animal (e.g., an elephant) and an object (e.g., a television), as well as a picture in which animal and object were presented together. The three pictures were recorded as video files and audio files were added to them. The animal movie lasted for 4 s and was paired with a sentence giving its name (e.g., “Look, the elephant.”). The object clip lasted for 4 s and was paired with a sentence describing the relationship between animal and object (e.g., “He likes to watch television.”). Finally, the combined movie lasted for 4 s and a sentence repeated the relationship (e.g., “The elephant likes watching television.”).

The stimulus material of the test phase consisted of the same 18 line drawings and additional items to examine children's recognition memory as well as their metacognitive judgments (see Table 1). The trigger item lasted for 6 s. It showed the animal drawing and was paired with a sentence triggering the object (“Do you still know what it likes to do?”). The test movie showed the animal on the left hand side and four possible answers on the right hand side presented vertically. This movie lasted for 6 s and was paired with the test question (“What does it like to do?”). A still frame of the test movie served as test item to assess children's answer to the question. Finally, the evaluation movie lasted for 6 s. It showed the animal on the left hand side and the CJ scale, which consisted of five smileys, on the right hand side. The evaluation clip was paired with a sentence asking for children's confidence (“How sure are you?”). The five smileys were vertically oriented and displayed a very happy smiley on the top and a sad looking smiley at the bottom. The three smileys in-between displayed gradual changes in the emotional valence. A still frame of the evaluation movie served as evaluation item to assess children's answer to the question.

**Table 1:** **List of items employed in the study**.

**Animal**	**Object**
Ape	Banana
Dog	Bone
Mouse	Cheese
Cat	Flower
Horse	Book
Duck	Pizza
Fish	Ice cream
Giraffe	Ball
Pig	Teddy
Owl	Swing
Elephant	Television
Cow	Chair
Snail	Candle
Fox	Bike
Rabbit	Car
Butterfly	Cake
Lion	Crayons
Bird	Lemonade

The first three items were used in the practice trials.

### Procedure

Children were tested individually in the laboratory and were seated in front of an eye-tracker. The experimenter explained to the child that she was going to watch a movie in which the child will hear stories about animals and their hobbies. All participants were tested at a viewing distance of approximately 60 cm from the monitor. During the experiment, the gaze of both eyes was recorded with a corneal reflection eye-tracker (Tobii T60, Tobii Technology, Sweden). The eye-tracking system was integrated in a 17-in (43.18 cm) TFT flat-screen monitor on which the stimuli were shown. The apparatus recorded gaze data at 60 Hz with an average accuracy of 0.5° visual angle. The movies were presented using the build-in software Tobii Studio (Tobii Technology, Sweden).

#### Practice trials

Three of the 18 items served as practice trials. In the practice trials, the experimenter sat next to the child and served as a model of how to perform the task. In a learning phase, the experimenter first watched the animal movie, the object movie, and the combined movie for every pair. In the test phase, the experimenter was first presented with the trigger item, before watching the test movie. When she was subsequently presented with the test item, she verbalized the task by saying: “Mhm, what did it like to do? I think the elephant liked to watch television. So, I need to point to the television here.” Then, she pointed to the television. Subsequently, she was presented with the evaluation movie and the evaluation item. Again, she verbalized the task by uttering: “Mhm, how sure am I?” For one item she told the child that she was sure of her answer and opted for one of the two smileys at the top. For one item the experimenter told the child that she was not sure and opted for one of the two smileys at the bottom, and for one item the experimenter told the child to be a bit sure and opted for the smiley in the middle.

#### Learning trials

The child was presented with the remaining 15 of the 18 items. For every pair, she first watched the animal movie, then the object movie, and finally the combined movie. No response was required from the participant. After every pair, an X appeared on the screen until the experimenter started the next trial by pressing the space bar.

#### Test trials

Learning trials and test trials were separated by a 10-min distractor task. In every trial, participants were first presented with a trigger item. Then, they watched the test movie. Subsequently, they were presented with the test item. The test item remained on the screen until the participants choose an object (i.e., verbally or by pointing to one of the objects) and the experimenter continued the study by pressing the space bar. Following the test item, participants watched the evaluation movie and were subsequently presented with the evaluation item. The evaluation item remained on the screen until the participants choose a smiley to indicate their level of confidence and the experimenter continued the study by pressing the space bar. Then, the next trial started.

### Measures

#### Object choice

We coded whether or not participants recognized the correct item.

#### Explicit confidence judgments

Participants' explicit CJs were coded by translating their judgments for the evaluation items into a scale ranging from 1 (low confidence) to 5 (high confidence).

#### Fixation time

To assess participants' implicit confidence in the evaluation phase, we analyzed their allocation of attention, reflected in their gaze fixations times, to the five smileys when watching the evaluation movie. We created same-sized areas of interest (AOIs) around the five smileys, each covering 1.86% of the area (ca. 24,000 pixels). The AOIs were not adjacent to each other, creating a space between them. We applied a standard fixation filter using a velocity threshold of 35 pixels/window and a distance threshold of 35 pixels to identify fixations. To calculate participants' fixations on the five smileys, we summed their total looking time on each smiley for the evaluation movie.

#### Pupil size

To analyze participants' pupil changes when presented with the trigger item, we assessed pupil size when watching the animal movie and when watching the trigger movie. To this end, pupil size was exported from Tobii Studio as a text file. MATLAB was used to process the data. For every participant, we calculated the average pupil size for the animal movie and the trigger movie.

## Results

### Object choice

Participants choose the correct item in 76% (*SE* = 2.3) of all trials.

### Explicit confidence judgments

For every participant we averaged their CJs for correctly and incorrectly remembered items. A *t*-test revealed that there was no difference for correctly (*M* = 3.98, *SE* = 0.26) and incorrectly (*M* = 4.10, *SE* = 0.21) remembered items, *t*_(11)_ = −0.609, *p* = 0.56.

### Fixation time

**Figure 1:**
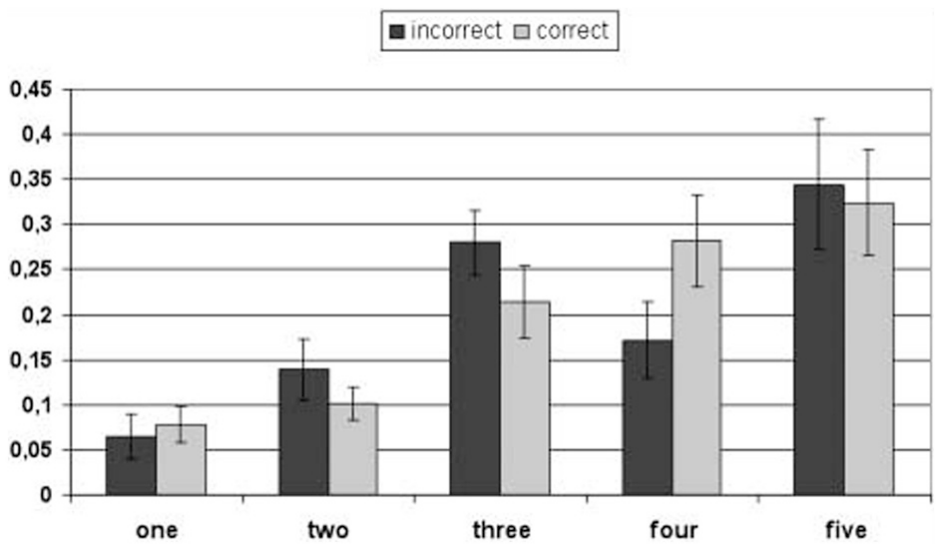
**Percentages of children's fixation times for the confidence scale (smileys one to five).** The relative fixation times are separately reported for correctly and incorrectly answered items. Error bars indicate standard errors of the means.

To analyze implicit CJs, participants' relative looking times on the five smileys were converted into percentage scores for every item. For every participant we calculated the average percentage looking time to each smiley for correctly and incorrectly remembered items by dividing the looking time to each smiley through the total looking time to all smileys. As for explicit CJs, the scale ranged from smiley 1 (low confidence) to smiley 5 (high confidence). Items for which no gaze on the smileys was recorded were discarded from analysis. On average, 13 items per child (*SE* = 0.59) were available for the analysis. Participants' average looking percentages were administered to a 2 (Item: correct vs. incorrect) × 5 (Smiley: 1–5) within-subjects analysis of variance (ANOVA). This analysis yielded a main effect of Smiley, *F*_(4, 44)_ = 5.507, *p* = 0.001, η^2^ = 0.33, which was modulated by an interaction between Item and Smiley, *F*_(4, 44)_ = 3.926, *p* < 0.01, η^2^ = 0.26 (for means see Figure 1). *Post-hoc* pairwise comparisons between correctly and incorrectly remembered items for each smiley revealed that participants looked longer at the fourth smiley for the correct than for the incorrect items, *p* < 0.01 (all other *p*s > 0.11). Moreover, the comparisons showed that for correct items looking times to smiley one differed from looking times to smileys three, four, and five, all *p*s < 0.01, and looking times to smiley two differed from looking times to smileys three, *p* < 0.05, four, and five, *p*s < 0.01 (all other *p*s > 0.21). For incorrect items, the comparisons revealed that participants looked relatively longer to smiley one than smileys two, *p* < 0.05, four, and five, *p*s < 0.01, and to smiley two than smiley three, *p* < 0.01 (all other *p*s > 0.05).

### Pupil size

To assess whether participants' pupil size changed from the baseline measurement during the learning phase (i.e., animal movie) to the test phase (i.e., trigger movie), we subtracted for every participant the average pupil size for the animal presentation in the learning phase (i.e., baseline assessment; animal movie) from the pupil size for exactly the same animal presentation in the test phase (i.e., trigger movie) for correct and incorrect items. On average, 14.25 items per child (*SE* = 0.35) were available for the analysis. One-sample *t*-tests against zero showed that there was a significant enlargement of pupil size for the correct items (*M* = 0.089, *SE* = 0.029), *t*_(11)_ = 3.039, *p* = 0.01, but no change for the incorrect items (*M* = 0.027, *SE* = 0.035), *t*_(11)_ = 0.79, *p* = 0.45.

## Discussion

The study aimed at investigating implicit metacognitive monitoring abilities in 3.5-year-old children by means of an eye-tracking paradigm. Children were presented with a paired picture associate learning task. In the test phase, their memory performance of the second picture was assessed when presented with the first picture. Subsequently, they were asked to give an explicit CJ of their memory performances while their fixation patterns at the confidence scale were recorded. The analyses show that participants' explicit CJ did not differ for correctly and incorrectly answered items. In contrast to the explicit judgments, participants' fixation pattern differed between the items. Moreover, an analysis of participants' pupil size revealed a change from learning to test phase for correctly answered items compared to incorrectly answered items. The results provide some evidence for implicit metacognitive memory monitoring processes in 3.5-year-old children and converge with other findings (e.g., Balcomb and Gerken, 2008; Lyons and Ghetti, 2013) in suggesting that implicit metacognitive abilities might precede the development of explicit metacognitive knowledge.

The explicit CJ revealed no differences for correctly compared to incorrectly answered items. This shows that on an explicit level children were not able to monitor their memory performances and could not differentiate between correct and incorrect answers. It should be noted that children's CJ were overall rather high (around 4 on a scale between 1 and 5). This finding replicates previous studies that showed on the one hand deficits in young children's explicit metamemory (e.g., Wellman, 1977; Lockl and Schneider, 2002) and on the other hand overconfidence in their own abilities and performances in young children (for a review see Schneider and Pressley, 1997).

In contrast, an analysis of looking behavior on the CJ scale revealed different fixation patterns for the correctly and incorrectly answered items. In particular, they showed that children tended to fixate on an item representing high confidence after having correctly answered an item than after having incorrectly answered an item. These findings extend behavioral studies with same aged children (Balcomb and Gerken, 2008; Lyons and Ghetti, 2013) and provide evidence for the presence of implicit metacognitive monitoring abilities in preschool children. The study relates to corresponding findings in Theory of Mind research, where earlier competencies in implicit measures (e.g., gaze behavior) have been reported than in explicit answers around 3 years of age (e.g., Clements and Perner, 1994). Our findings thereby support theoretical accounts that suggest that metacognitive monitoring need not depend on explicit knowledge about cognitive processes, but can be based on experience-based cues during the actual learning process (Koriat, 1995, 2007).

Yet, it should be noted that the effect was restricted to the middle range of the confidence scale. In particular we found no difference in looking time for the smileys denoting the highest and the lowest CJs. This corresponds partly to the findings of Roderer and Roebers (2010) who reported no differences between easy, difficult, and unanswerable items for the lowest part of the confidence scale. It is possible that a bias for high confidence ratings or a bias against low confidence ratings (also present in children's explicit judgments) overruled children's differential allocation of fixation time to the lower confidence ratings. As a consequence, the scale could only differentiate in its middle range. Alternatively, the result could suggest that implicit metacognitive knowledge is still rather weak at the preschool age. For future studies, it might be interesting to employ a scale that allows for a better differentiation (e.g., by using 9 instead of 5 gradations).

Interestingly, measures of pupil size showed an enlargement of pupil size when children were presented with an item they were able to answer correctly (compared to a baseline assessment). This was not the case for incorrectly answered items, indicating that pupil changes differentiated between correctly and incorrectly remembered items. It should be noted that this finding cannot be explained by different pupil size for different items (e.g., driven by differences in illumination). Given that we compared pupil size to exactly the same items in a baseline period and in the test phase, the findings cannot be due to perceptual differences between remembered and non-remembered items.

Our results show enlarged pupil size for correctly, but not incorrectly remembered items. This relates to recent studies with adult populations (Papesh et al., 2011) and suggests that also in preschool children changes in pupil size reflect implicit or preconscious cognitive processes (cf. Gredebäck and Melinder, 2011; Laeng et al., 2012). The results could be interpreted in two ways. On the one hand, given that pupil dilations were found for the trigger items (i.e., before participants were presented with the test movies) and given recent findings of a relationship between pupil size during memory retrieval and subsequent confidence ratings (Papesh et al., 2011), our results could point to implicit abilities in preschool children to evaluate their performance in a memory task (i.e., knowledge that they will or will not be able to remember the correct items). On the other hand, given that pupil sizes were compared for correct vs. incorrect items and not directly for high-confidence vs. low-CJs, it is also possible that they merely reflect memory processes (such as retrieval) rather than metacognitive processes. Future research is needed to investigate the role of pupil changes in preschooler's memory processes in greater detail.

The present study supports eye-tracking as a suitable method to examine cognitive performances in young children (for a review see Gredebäck et al., 2010). It extends findings on metacognitive monitoring from school-aged children to preschoolers (Roderer and Roebers, 2010) and relates to studies employing eye-tracking to reveal young children's expectations and knowledge (e.g., McMurray and Aslin, 2004; Paulus, 2011; Paulus et al., 2011; Daum et al., 2012; Elsner et al., 2012a,b). Notwithstanding the insight gained from the present work, it is worth to point to some limitations of the study. First, it would be interesting to examine even younger children and to investigate whether they already show signs of implicit metacognitive monitoring abilities. The current study design relies on verbal instruction in the memory task and is thus not suitable to conduct with children below the age of 3. A modified design is needed to examine the early roots of implicit metacognitive abilities in younger children (Sodian et al., 2012). Moreover, the current results provide only weak evidence for implicit metacognitive abilities in 3.5-year-old children as the effect was restricted to the middle range of the confidence scale (i.e., smileys 3 and 4). Further research is thus needed to clarify whether this finding is due to methodological limitations or rather indicative for weak implicit metacognitive abilities in 3.5-year-old children. Furthermore, on a theoretical level the relation between implicit and explicit metacognitive abilities needs further clarification (for discussions see Carruthers, 2009; Perner, 2012; Proust, 2010, 2013). In particular, it would be of pivotal importance to examine longitudinally whether the implicit measures as assessed in this study relate to later explicit metacognitive knowledge. Such relations have been reported, for example, for the development of social-cognitive skills (e.g., Wellman et al., 2008; Thoermer et al., 2012).

In conclusion, the present study examined early signs of metacognitive monitoring in preschoolers by means of an eye-tracking paradigm. The findings provide some evidence for implicit metacognitive skills in 3.5-year-old children. It converges with recent behavioral evidence (Balcomb and Gerken, 2008; Gerken et al., 2011; Lyons and Ghetti, 2013) that implicit signs of metacognitive monitoring might precede their explicit manifestations in verbal tasks.

### Conflict of interest statement

The authors declare that the research was conducted in the absence of any commercial or financial relationships that could be construed as a potential conflict of interest.
